# Relax, Keep Walking — A Practical Guide to Continuous Phylogeographic Inference with BEAST

**DOI:** 10.1093/molbev/msab031

**Published:** 2021-02-02

**Authors:** Simon Dellicour, Mandev S Gill, Nuno R Faria, Andrew Rambaut, Oliver G Pybus, Marc A Suchard, Philippe Lemey

**Affiliations:** 1 Spatial Epidemiology Lab (SpELL), Université Libre de Bruxelles, Bruxelles, Belgium; 2 Department of Microbiology, Immunology and Transplantation, Rega Institute, KU Leuven, Leuven, Belgium; 3 MRC Centre for Global Infectious Disease Analysis, J-IDEA, Imperial College London, London, United Kingdom; 4 Department of Zoology, University of Oxford, Oxford, United Kingdom; 5 Instituto de Medicina Tropical, Faculdade de Medicina da Universidade de São Paulo, São Paulo, Brazil; 6 Institute of Evolutionary Biology, University of Edinburgh, Edinburgh, United Kingdom; 7 Department of Biomathematics, David Geffen School of Medicine, University of California, Los Angeles, Los Angeles, CA, USA; 8 Department of Biostatistics, Fielding School of Public Health, University of California, Los Angeles, Los Angeles, CA, USA; 9 Department of Human Genetics, David Geffen School of Medicine, University of California, Los Angeles, Los Angeles, CA, USA

**Keywords:** relaxed random walk, continuous phylogeography, viruses, BEAST

## Abstract

Spatially explicit phylogeographic analyses can be performed with an inference framework that employs relaxed random walks to reconstruct phylogenetic dispersal histories in continuous space. This core model was first implemented 10 years ago and has opened up new opportunities in the field of phylodynamics, allowing researchers to map and analyze the spatial dissemination of rapidly evolving pathogens. We here provide a detailed and step-by-step guide on how to set up, run, and interpret continuous phylogeographic analyses using the programs BEAUti, BEAST, Tracer, and TreeAnnotator.

## Introduction

**Fig. 1.**
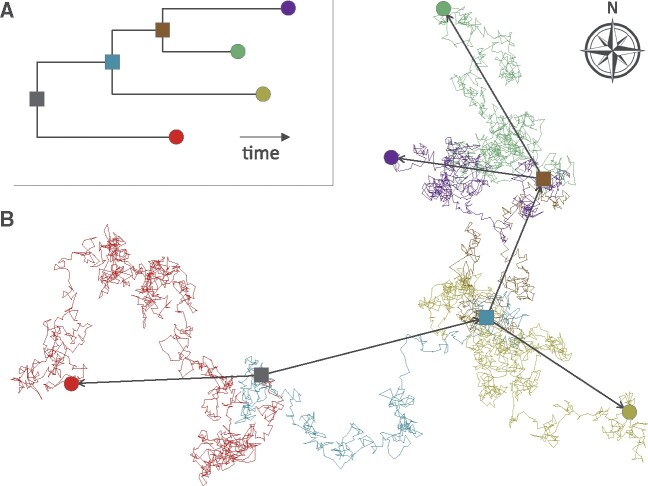
Link between inferred phylogeny (*A*) and inferred dispersal history of phylogenetic branches (*B*; inspired by Pybus et al. [2012] and Holbrook et al. [2020]). Filled circles represent sampled sequences for which locations and dates of sampling are known. Squares represent unsampled ancestral nodes for which locations and dates are estimated. Branch lengths in (*A*) reflect the time elapsed in each lineage and thin colored lines in (*B*) show the RRW (i.e., allowing branch-specific variation in dispersal velocity) undertaken by each lineage. In (*B*), straight arrows indicate the direction and distance of the movement trajectory defined by each lineage.

Bayesian Evolutionary Analysis by Sampling Trees (BEAST) (Suchard et al. 2018) is one of the most widely used software packages for Bayesian phylogenetic inference. The ability to perform phylogeographic reconstruction has at least partly contributed to its popularity. BEAST offers both discrete and continuous phylogeographic approaches. The first approach (Lemey et al. 2009) uses a continuous-time Markov chain to model the movement of viral lineages among a set of discrete locations, whereas the second approach (Lemey et al. 2010) exploits a two-dimensional relaxed random walk (RRW) to reconstruct viral dispersal history in continuous space. Inspired by uncorrelated relaxed clock models (Drummond et al. 2006), the development of the RRW model allows branch-specific variation in dispersal velocity (Pybus et al. 2012; Holbrook et al. 2020), which provides more flexibility as compared with a standard Brownian diffusion model. In practice, it means that in the RRW model, dispersal velocity can vary across the tree but remains constant along each branch. As with the discrete approach implemented in BEAST, continuous phylogeographic analyses involve a joint inference of both the phylogenetic tree (representing the evolutionary relationships between sampled sequences) and the geographic locations of unsampled common ancestors (fig. 1), thereby producing a spatially explicit history of dispersal of the sampled population (Faria et al. 2011).

The continuous approach to phylogeography in BEAST remains less frequently used than its discrete counterpart, even though the latter can present several methodological disadvantages (Dellicour, Vrancken, et al. 2018). First, the discrete phylogeographic method often requires an arbitrary grouping of sampling locations that may lead to oversimplified abstraction or unrealistic subdivision of the study area. Second, heterogeneous sampling effort or sampling bias can severely compromise discrete phylogeographic reconstructions (De Maio et al. 2015; Baele et al. 2017). Under- or oversampling efforts can affect the estimates of transition rates between sampled locations and hence also the phylogeographic reconstruction. However, although the impact of heterogeneous sampling effort has mostly been analyzed for discrete models, Kalkauskas et al. (2020) have recently investigated how it can affect continuous phylogeographic reconstructions. Their results confirm that continuous models can also be affected by the lack of sampling in certain areas (Kalkauskas et al. 2020). Finally, the restriction that the locations of all ancestors of the sampled viruses can only correspond to the sampled locations in the discrete approach can limit the realism of the inferred dispersal history. For these reasons, the continuous phylogeographic approach can provide a more realistic alternative.

The less frequent application of the continuous phylogeographic model could in part be due to the difficulty of obtaining sampling geographic coordinates for all sequences of interest, the challenges of modeling dispersal as a continuous process (Faria et al. 2011), or lower familiarity with the installation and interpretation of a continuous diffusion model. To remedy the latter issue, we describe here how to prepare and run such a continuous phylogeographic analysis. For illustration, we provide a step-by-step protocol based on the analysis of a data set of yellow fever virus (YFV) genomic sequences sampled during one of the largest YFV epidemics in Brazil (Faria et al. 2018).

### Data Set Description

This protocol provides a detailed guide for reconstructing the spatial dynamics of the YFV epidemic in Brazil through the analysis of a set of viral genome sequences that were sampled at different points in time (Faria et al. 2018). YFV is responsible for 29,000–60,000 deaths annually in South America and Africa and is the most severe mosquito-borne infection in the tropics. Recently, Brazil experienced its largest recorded yellow fever outbreak in decades. In that context, Faria et al. (2018) analyzed a data set of 65 YFV genomes collected between January and April 2017 in order to characterize the dispersal history and viral transmission dynamics of this outbreak. The continuous phylogeographic method implemented in BEAST was used to estimate the ancestral YFV locations in continuous space.

## Protocol

### Step 1: Using BEAUti to Set Up the BEAST Analysis

**Fig. 2.**
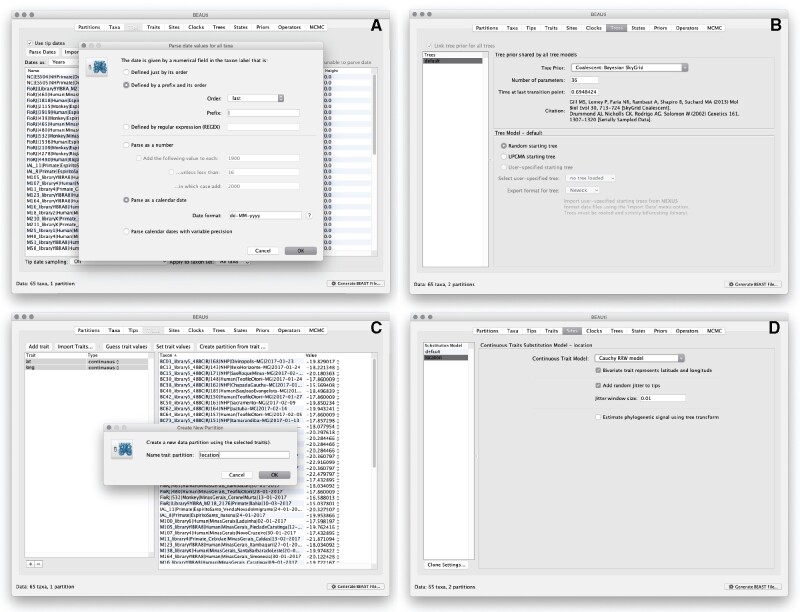
Setting up the continuous phylogeographic analysis in BEAUti: specifying the sampling date associated with each sampled sequence (*A*), setting up the coalescent model (*B*), entering the sampling coordinates (*C*), and specifying the RRW model (*D*). Zoomed versions of these figures can be found in our detailed online protocol: https://beast.community/workshop_continuous_diffusion_yfv.

The program BEAUti is distributed in the BEAST package and is a user-friendly interface for specifying model and Markov chain Monte Carlo (MCMC) settings for a BEAST analysis. The sequence alignment is first uploaded by selecting the **Import Data** option from the **File** menu. Here, we load an alignment of 65 YFV genomes 10,236 nucleotides in length. BEAST analyses that model evolution according to a single bifurcating tree assume the absence of recombination events within the genomic sequence alignment under consideration. Recombination can be detected through the Φ-test (Bruen et al. 2006) or using RDP (Martin et al. 2015) for example, and can sometimes be filtered from the data. Once loaded, the sequence data are listed under the **Partitions** panel and we next specify the sampling date information, which starts by selecting the box labeled **Use tip dates** in the **Tips** panel (fig. 2*A*). By default, all taxa are assumed to have a date of zero, which means that sequences are assumed to be sampled at the same time. That is not the case for the YFV sequences which were sampled on different days during 2017. The sampling time in years is encoded in the name of each taxon and we could simply edit the value in the **Date** column of the table to reflect these. However, if the taxa names contain the calibration information, then a convenient way to specify the dates of the sequences in BEAUti is to use the **Parse Dates** button at the top left of the **Tips** panel. Clicking this will make a dialog box appear (fig. 2*A*). This operation facilitates the extraction of date information contained within the taxon names: it works by trying to find a numerical field within each name. If the taxon names contain more than one numerical field, then we can specify how to find the one that corresponds to the date of sampling. We can 1) specify the order that the date field comes (e.g., first, last, or various positions in between), 2) specify a prefix (some characters that come immediately before the date field in each name) and the order of the field, or 3) define a regular expression (REGEX). There is also an option to parse calendar dates with various precisions. For the YFV sequences, dates values have to be parsed as calendar dates by specifying the format “dd-MM-yyyy.” The sampling dates will appear in the appropriate column of the main window. In addition, the Height column will list the ages of the tips relative to time 0 (in our case 2007.63). Finally, the Uncertainty column allows us to specify with what precision the sampling time is known. This is not useful in our case because all sampling dates are known exactly, but BEAST allows us to integrate over uncertainty in sampling dates (e.g., when only the sampling month or year is known).

We now move on to the **Trees** panel, to set the **Tree Prior** that describes how population size is expected to change over time according to a coalescent model (fig. 2*B*). The default tree prior is set to a constant size coalescent prior. Here, we will select the flexible skygrid coalescent model (Gill et al. 2013; Hill and Baele 2019) as demographic tree prior (**Coalescent: Bayesian SkyGrid**), with 36 grid points (**Number of parameters**) and a time at last transition point set to 0.6948424. By doing so, the grid points approximate the number of epidemiological weeks spanned by the duration of the phylogeny (Faria et al. 2018).

The next step is to click on the **Traits** panel at the top of the main window (fig. 2*C*). A trait can be any characteristic that is inherent to the specified taxon, for example, geographical location or host species. Here, we will assign a latitude and longitude as bivariate geographical location to each taxa based on the trait specification for each sequence. To associate the sequences with the traits, we need to click on **Import Traits**, which will open a new window that allows us to import a file with the trait values, that is, tab-delimited file that links each taxon with its sampling coordinates (latitude and longitude). After the import, we have to select both the latitude and longitude traits in the left window and click on **Create partition from trait**. In the window that pops up, we can then enter a name for this partition, for example, “location.” The resulting new partition with two **Sites** and a continuous **Data Type** will be shown under the **Partitions** panel.

Next, we click on the **Sites** panel to set the sequence and trait evolutionary models. For the **Nucleotide Substitution Model**, we will here keep the default **HKY** substitution model and the **Base frequencies** to be **Estimated**, specify the **Site Heterogeneity Model** to **Gamma**, and keep the default **Partition into codon** positions to **Off**. We subsequently set the substitution model for the **location** partition by selecting the **Cauchy RRW model** and by specifying that **Bivariate trait represents latitude and longitude** (fig. 2*D*). The latter option allows estimating diffusion statistics that are specific for bivariate spatial traits (with latitude and longitude in that order). “Cauchy” refers to the name of the probability distribution that is here used to accommodate dispersal velocity variation among phylogeny branches. We also select the option **Add random jitter to tips**, which adds noise to sampling coordinates. With this option, the noise is drawn uniformly at random from a particular **Jitter window size** to duplicated (location) traits. Here, we set the jitter window size to 0.01, which will add a small noise that will avoid a poor performance of the RRW model when not all sequences are associated with unique sampling coordinates. The choice of the jitter value is arbitrary, but it should remain sufficiently small to avoid alternating too much the actual geographic origin of each sample (see also our discussion below about alternatives to the jitter option).

The **Molecular Clock Model** can be subsequently set in the **Clocks** panel where we can choose between a strict and a relaxed (uncorrelated lognormal or uncorrelated exponential) clock model. We will perform our analysis using the **Uncorrelated relaxed clock** model with an underlying **Lognormal** distribution. In the **States** panel, we only need to check that the location partition is set to **Reconstruct states at all ancestors** (default option). In this case, there is no need to change the default parametrization in the **Priors** and **Operators** panels, but further detail on this setting can be found in the detailed tutorial for this protocol (see the Data Availability section).

The **MCMC** panel in BEAUti provides settings to control the MCMC chain. We can first define the **Length of the chain**, which is the number of steps the MCMC will make in the chain before finishing. The length of the chain should depend on the size of the data set, the complexity of the model, and the precision of the answer required. The default value of 10,000,000 is entirely arbitrary and should be adjusted according to the size of the considered data set. As described below, the resulting log file can be analyzed using the program Tracer in order to examine whether a particular chain length is adequate. The next couple of options specifies how often the current parameter values should be displayed on the screen (**Echo state to screen every**) and recorded in the log file (**Log parameters every**). The screen output is simply for monitoring the program’s progress and can therefore be set to any value (although if set too small, the sheer quantity of information being displayed on the screen will slow the program down). For the log file, the value should be set relative to the total length of the chain. Sampling too often will result in very large files with little extra benefit in terms of the precision of the estimates. Sample too infrequently will result in the log files containing insufficient information about the distributions of the parameters. Overall, we aim to store no more than 10,000 sampled states. For the present data set, we will set the **Length of the chain** to 500,000,000 and the parameter **Log parameters every** as well the **Echo state to screen every** 50,000 states.

The next option in the **MCMC** panel allows the user to set the **File name stem** which is here set to “YFV_RRW_cauchy” (for “relaxed random walk Cauchy diffusion model”). The next two options set the file names of the log files for the parameters and the trees but are by default based on the **File name stem**. Finally, one can select to perform marginal likelihood estimation to assess model fit (Baele et al. 2016), which is not needed in this exercise. At this point, we are ready to generate a BEAST XML file and to use this to run the Bayesian evolutionary analysis. We can do this by clicking on the button labeled **Generate BEAST File…** at the bottom of the window.

### Step 2: Performing the Analysis in BEAST

Once the input XML file has been created, we launch BEAST to perform the analysis itself. The exact instructions for running BEAST depends on the computer used, but in most cases a dialog box will appear in which we can select the XML file. We can also launch BEAST by using its command line version. In that case, the name of the XML file is specified after the name of the BEAST executable and detailed information about the progress of the analysis will be written to the terminal. When it has finished, the log file and the trees file will have been created in the same location as your XML file. It is important to note that BEAST requires the installation and use of the BEAGLE library (https://github.com/beagle-dev/beagle-lib), which enables fast likelihood computation via parallel computing (Ayres et al. 2019).

### Step 3: Using Tracer to Assess Convergence and Mixing

**Fig. 3.**
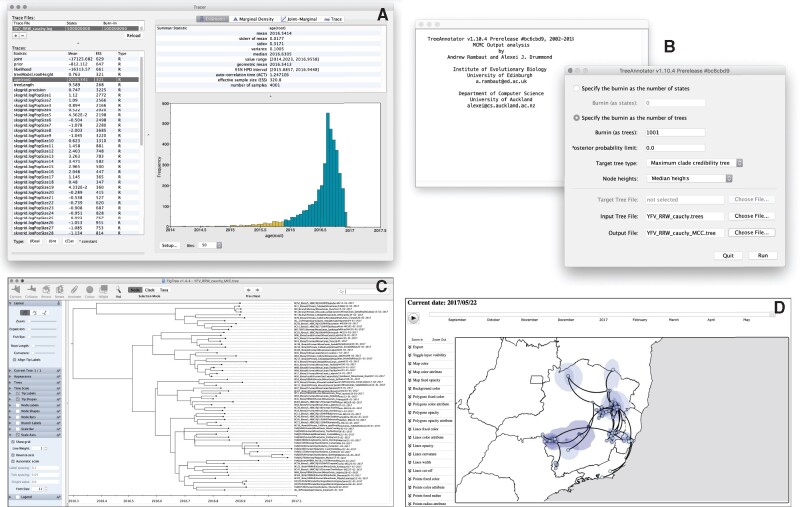
Analyzing the outputs of the continuous phylogeographic inference: assessing convergence and mixing with Tracer (*A*), summarizing the posterior trees by using TreeAnnotator to find and annotate the MCC tree (*B*), visualizing the resulting MCC tree with FigTree (*C*), and using spreaD3 to visualize the phylogeographic reconstruction (*D*). Zoomed versions of these figures can be found in our detailed online protocol: https://beast.community/workshop_continuous_diffusion_yfv.

We employ the user-friendly program Tracer (Rambaut et al. 2018) to analyze the results of the BEAST run (fig. 3*A*). We first use the **Import Trace File…** option from the **File** menu of Tracer to load the log file generated by BEAST (here called “YFV_RRW_cauchy.log”). On the left-hand side is the name of the log file loaded and the traces that it contains. Selecting a trace on the left brings up various summary analyses of this trace on the right-hand side, according to which tab is selected. In the top right of the window, there is a table of summary statistics for the selected trace, for example, the **mean** and **median** values of the parameter states excluding the burn-in, the 95% highest posterior density (HPD) interval (**95% HPD interval**), **the auto-correlation time (ACT)** defined as the average number of states in the MCMC that two samples have to be separated by for them to be uncorrelated (i.e. independent samples from the posterior, and the **effective sample size (ESS)**, which is the chain length (excluding the burn-in) divided by the ACT, and represents the number of independent samples that the trace is equivalent to.

When the ESS values are small (ESS values <100 are highlighted in red by Tracer and values >100 but <200 are in yellow), it indicates that the posterior sample is equivalent to relatively few independent samples and thus may not accurately represent the posterior distribution. A simple strategy to remedy this problem is to run the MCMC chain for longer, until sufficient ESS values are achieved. Largely independent of the use of the RWW model, the runtimes of BEAST analyses are highly dependent on the size of the data set, both in terms of number of samples and genomic sequence length, and can range from a few hours to several days or even longer. For instance, the analysis of the YFV data set used as an example required 5 × 10^8^ iterations to achieve sufficient ESS values (i.e., all ESS values >200). This corresponded to a runtime of approximately 30 h on a MacBook Pro 3.1 GHz Quad-Core Intel Core i7.

### Step 4: Summarizing the Posterior Tree Distribution

BEAST samples time-scaled trees as well as the model parameters. The trees are written to a separate NEXUS format file with a “.trees” extension (here “YFV_RRW_cauchy.trees”). The program TreeAnnotator is provided as part of the BEAST package and can be used to summarize the information within our sampled trees (fig. 3*B*). TreeAnnotator takes a single “target” tree and annotates it with the summarized information from the entire sample of trees, such as average node ages (along with their HPD intervals) and the posterior support and the average rate of evolution on each branch (for relaxed clock models). Prior to running Tree Annotator, several options have to be specified: the number of steps in the MCMC chain (**Burnin as states**), or the number of trees (**Burnin as trees**) that should be excluded from the summarization, the minimum posterior probability for a node in order for TreeAnnotator to store the annotated information (**Posterior probability limit**), the Target tree type (see below), the **Node heights** specifying what node heights (times) should be used for the output tree, the **Target Tree File** to select a NEXUS file containing the target tree (when the **User target tree** option is selected, which is not the case here), the posterior distribution of trees to consider (**Input Tree File**), and the name for the output tree file (e.g. “YFV_RRW_cauchy_MCC.tree”).

We keep the default **Posterior probability limit** set to 0.0 so every node, no matter what its support, will have its information summarized. For the **Target Tree File**, there are two options: **Maximum clade credibility tree** (MCC tree) or **User target tree**. We here select the first option so that TreeAnnotator will examine every sampled tree and select the tree with the highest product of the posterior probabilities across all of its nodes. We will also select the **Keep target heights** option to keep node heights as in the selected tree, but note that node heights can also be summarized as a mean or a median over the sample of trees. In the latter case, however, a mean or median height for a node may sometimes be higher than the mean or median height of its parental node (because particular ancestral-descendent relationships in the MCC tree may still be different compared with a large number of other sampled trees), resulting in artifactual negative branch lengths.

The tree generated by TreeAnnotator is in standard NEXUS tree file format that can be loaded into any tree drawing package that supports this. However, it also contains additional information that can only be displayed using the FigTree program (fig. 3*C*). On the left-hand side of the FigTree window are the options and settings which control how the tree is displayed. In the Layout panel, we can, for instance, not only select the check-box **Align Tip Labels** to increase clarity but also plot a time scale axis for this evolutionary history (by selecting **Scale Axis** and deselecting **Scale Var**).

### Step 5: Visualizing the Continuous Phylogeographic Reconstruction

The program spreaD3, which stands for “Spatial Phylogenetic Reconstruction of EvolutionAry Dynamics using Data-Driven Documents” (D3), can be used to visualize the output from continuous phylogeographic inference (Bielejec et al. 2016). It is a user-friendly application to analyze and visualize phylodynamic reconstructions resulting from Bayesian molecular clock inference of sequence and trait evolutionary processes. This program also allows for visualization on custom maps and generates HTML pages for display in browsers, such as Firefox, Safari, and Chrome. One of the functions of spreaD3 that relates to continuous phylogeographic analysis is the visualization of location-annotated MCC trees (fig. 3*D*). A detailed tutorial for this particular step can be found on the program website (https://rega.kuleuven.be/cev/ecv/software). A second option is to use the R functions implemented in the package “seraphim” (Dellicour, Rose, Faria, et al. 2016) to map the MCC and associated credible intervals onto customized maps. A detailed tutorial for using “seraphim” to map the outcome of the continuous phylogeographic inference performed in this protocol can be found on the corresponding GitHub repository (https://github.com/sdellicour/ seraphim).

## Related Models and Methods

Since its initial implementation, several additional models for continuous phylogeography and trait evolution have been implemented in BEAST, with similar steps for carrying out analyses. Notably, the relaxed *directional* random walk model extends the RRW model by accommodating directional trends in spatial diffusion that can vary along the phylogenetic tree (Gill et al. 2017). This model has, for instance, been used to infer the phylogeographic history of the porcine deltacoronavirus while accommodating latitudinal (South–North) drift of its spread in China (He et al. 2020).

A common issue in continuous phylogeography is dealing with sampling locations that are unavailable or not known with sufficient precision. Although the RRW model conditions on unique sampling coordinates for each sample, publicly available sequences are frequently only associated with relatively large administrative regions (or even countries). In such situations, polygons can be defined and used to specify prior ranges of sampling coordinates. This can be done by defining a uniform sampling probability within the unique polygon associated with a sampled sequence (Nylinder et al. 2014) or alternatively by using prior knowledge in order to specify heterogeneous sampling probabilities over a collection of subpolygons assigned to each sequence (Dellicour et al. 2020). In the latter approach, external data, such as outbreak locations or host species distributions, can be used to subdivide the administrative area of origin into a collection of subpolygons associated with different sampling probabilities. These polygons can also be used as an alternative to the default “jitter” option (introduced above) that is used to avoid duplicate sampling coordinates. When several sequences share the exact same sampling coordinates (e.g., when sequences are sampled from the same city and all assigned a location corresponding to the city’s centroid), this option adds uniformly drawn noise from a user-defined window to location coordinates. However, using the jitter option to add noise may present problems such as sampling coordinates for pathogens with terrestrial hosts falling in areas of water (Dellicour, Baele, et al. 2018).

Finally, a range of post hoc analyses for continuous phylogeography have been implemented in the R package “seraphim” (Dellicour, Rose, and Pybus 2016; Dellicour, Rose, Faria, et al. 2016). These include extraction of the spatiotemporal information embedded in posterior trees obtained by continuous phylogeographic inference, estimation of dispersal statistics (e.g., lineage dispersal velocity, diffusion coefficient, evolution of the maximal wavefront distance), as well as investigation of the impact of environmental factors on the dispersal velocity (Dellicour et al. 2017), frequency (Dellicour, Baele, et al. 2018), and position (Dellicour et al. 2019) of viral lineages. The package “seraphim” can also be used to generate graphical representations of continuous phylogeographic reconstructions (Faria et al. 2018; Rakotomalala et al. 2019; Candido et al. 2020).

The RRW model has been recently applied in numerous phylogeographic analyses aimed at understanding the dispersal history of viruses responsible for notable epidemics, such as bluetongue virus (Jacquot et al. 2017), Ebola virus (Dellicour, Baele, et al. 2018), HIV (Faria et al. 2019), foot-and-mouth disease virus (Duchatel et al. 2019), and Lassa virus (Ehichioya et al. 2019). Although the development of the RRW model was primarily motivated by phylogeographic applications, it has also been frequently used to study the evolutionary history of other continuous traits such as phenotypic measures (Bedford et al. 2014; Hassler et al. 2020; Monjane et al. 2020; Zhang et al. 2020).
